# Atropine-Induced Anticholinergic Syndrome Following Off-Label Sublingual Administration for Refractory Sialorrhea: A Case Report

**DOI:** 10.7759/cureus.113928

**Published:** 2026-08-04

**Authors:** Pedro Reboredo, Paula Nogueira, Rita Rosa Domingos, Cristina Sousa, Ramiro Lopes

**Affiliations:** 1 Internal Medicine, Unidade Local de Saúde do Algarve - Hospital de Faro, Faro, PRT

**Keywords:** anticholinergic syndrome, atropine, delirium, head and neck cancer, medication administration error, off-label atropine, patient safety, sialorrhea

## Abstract

A sublingual atropine ophthalmic solution is increasingly used as an off-label therapy for the symptomatic management of refractory sialorrhea because of its rapid onset of action, ease of administration, and generally favourable safety profile when used at recommended doses. Although generally well-tolerated at therapeutic doses, systemic anticholinergic toxicity may occur following medication administration errors or unintentional overdose.

We report the case of a 76-year-old man with recurrent head and neck squamous cell carcinoma receiving palliative systemic therapy who presented with acute hyperactive delirium, severe psychomotor agitation, bilateral mydriasis, diffuse cervicothoracic flushing, tachycardia, and severe hypertension. Careful medication reconciliation revealed inadvertent repeated administration of an unquantified number of sublingual atropine drops by a visually impaired caregiver instead of the prescribed regimen. Alternative infectious, metabolic, and medication-related causes were excluded, and the patient was diagnosed with atropine-induced anticholinergic syndrome. Prompt discontinuation of atropine, supportive care, and intravenous neostigmine resulted in complete clinical recovery within 14 hours.

This case highlights the importance of recognising the characteristic anticholinergic toxidrome, performing meticulous medication reconciliation, and considering caregiver-related factors when prescribing off-label therapies that require precise dose administration. Careful assessment of the patient's and caregiver's ability to administer medication safely is essential to prevent avoidable adverse drug events.

## Introduction

Sialorrhea is a common and distressing symptom in patients with neurological disorders and advanced head and neck cancer, primarily resulting from impaired swallowing and reduced clearance of oral secretions rather than excessive saliva production [1-3]. Persistent sialorrhea may substantially impair quality of life, increase caregiver burden, and contribute to complications such as aspiration, peristomal skin breakdown, and recurrent respiratory infections [2,3]. When conservative measures fail, pharmacological treatment is frequently required, with antimuscarinic agents, including glycopyrrolate, hyoscine, and atropine, representing commonly used therapeutic options [1-3].

Among these, sublingual administration of atropine ophthalmic solution has gained increasing acceptance as off-label therapy because of its rapid onset of action, ease of administration, and widespread availability [4-9]. Although generally considered safe at therapeutic doses, systemic absorption may result in clinically significant anticholinergic toxicity, particularly when administration errors or excessive dosing occur [10,11]. However, reports of systemic anticholinergic toxicity following off-label sublingual atropine administration remain scarce [10], particularly those highlighting medication administration errors and caregiver-related factors.

We report the case of a patient with recurrent head and neck cancer receiving palliative treatment who developed severe anticholinergic syndrome following inadvertent sublingual atropine overdose caused by a medication administration error. In addition to describing a rare adverse drug event, this case highlights the importance of recognising the characteristic anticholinergic toxidrome, performing meticulous medication reconciliation, and assessing caregiver-related factors when prescribing off-label therapies that rely on precise dose administration.

## Case presentation

A 76-year-old man presented to the emergency department with a 4-hour history of acute psychomotor agitation and altered mental status. His medical history was notable for recurrent head and neck squamous cell carcinoma previously treated with extensive surgery and adjuvant chemoradiotherapy, resulting in a permanent tracheostomy. At the time of presentation, he was receiving palliative systemic therapy for unresectable locoregional recurrence. His past medical history also included low rectal adenocarcinoma treated with neoadjuvant chemoradiotherapy with complete clinical response, primary hypertension, and chronic cancer-related pain. His regular medications comprised ramipril, tramadol/paracetamol as needed for cancer-related pain, lorazepam as needed for insomnia, and atropine ophthalmic solution (10 mg/mL), prescribed as one sublingual drop every 12 hours as needed for refractory sialorrhea. He had no known drug allergies.

**Figure 1:**
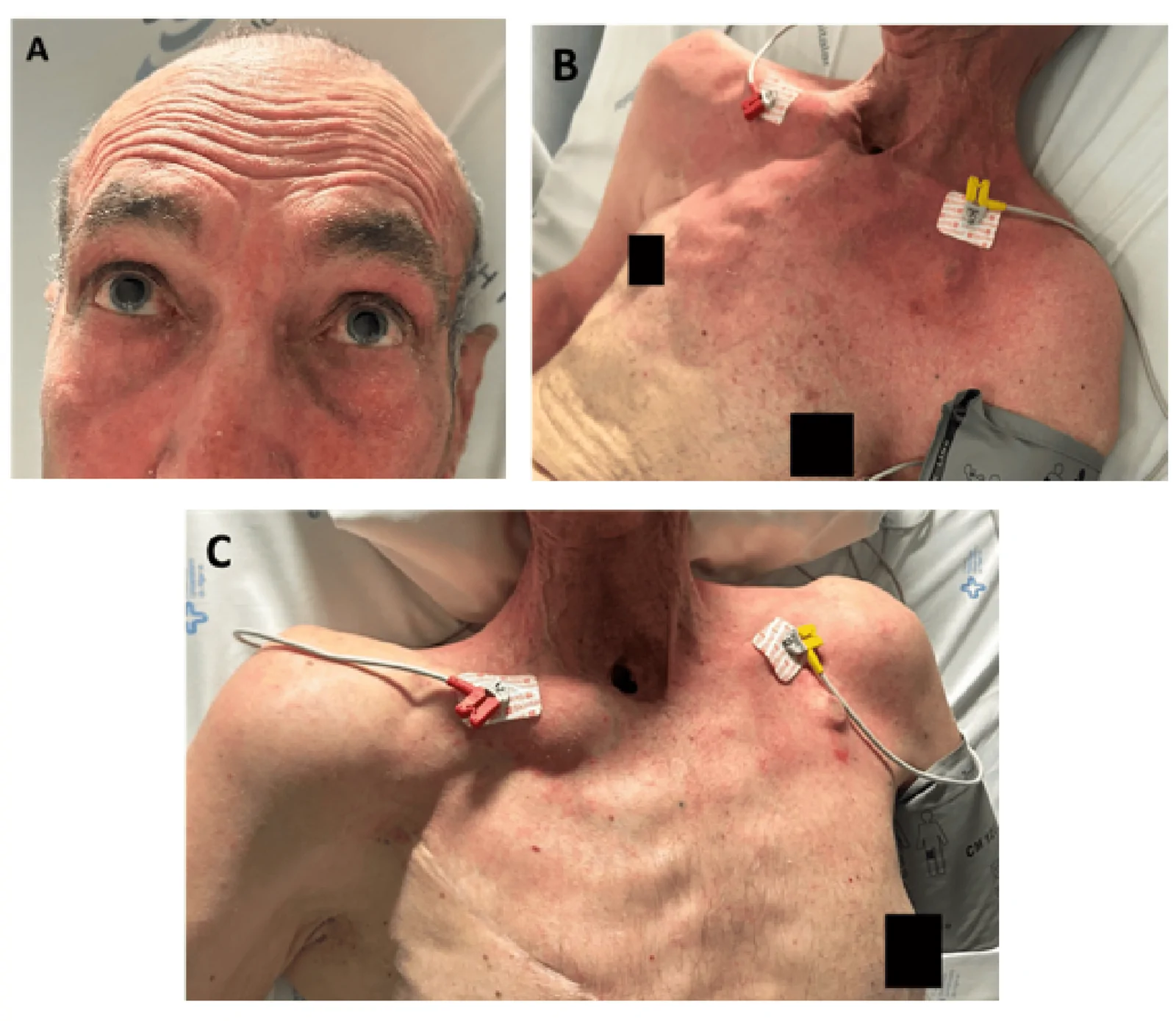
Clinical manifestations of atropine-induced anticholinergic syndrome before and after treatment 1A: Marked bilateral mydriasis and diffuse facial flushing on presentation; 1B: Extensive flushing involving the neck and upper chest on admission; 1C. Partial resolution of the cutaneous flushing after initiation of treatment

On arrival, the patient was severely agitated, disoriented, and combative. His blood pressure was 198/100 mmHg, heart rate was 163 beats/min, and body temperature was 36.5 °C. Physical examination revealed marked bilateral mydriasis, diffuse flushing involving the face, neck, and upper trunk, and hot, dry skin (Figures 1A, 1B).

**Figure 2:**
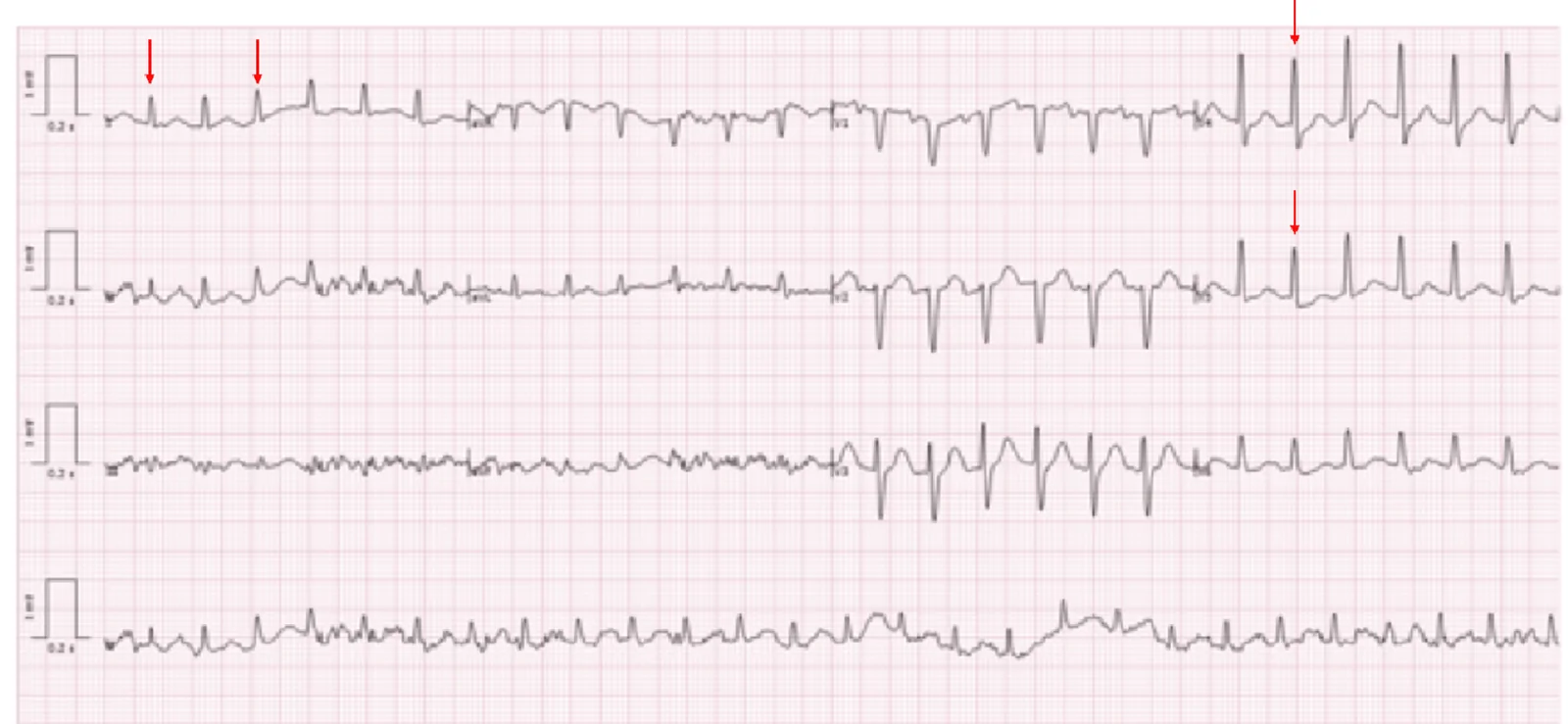
Initial 12-lead electrocardiogram Regular narrow-complex tachycardia at 163 beats/min. The arrows indicate representative narrow QRS complexes. The automated corrected QT interval was 494 ms.

Cardiopulmonary and abdominal examinations were otherwise unremarkable. Initial 12-lead electrocardiography demonstrated a narrow-complex supraventricular tachycardia at 163 beats/min with a corrected QT interval (QTc) of 494 ms (Figure 2).

Initial laboratory investigations revealed mild neutrophilic leukocytosis without significant metabolic abnormalities or evidence of an alternative cause for the patient's presentation. Laboratory investigations are summarised in Table 1.

**Table 1:** Initial laboratory investigations The table lists the results of the patient's initial laboratory investigations, including complete blood count, leukogram, and selected biochemical parameters. Mild neutrophilic leukocytosis was the only clinically relevant abnormality. Reference ranges correspond to the institutional laboratory.

Laboratory parameter	Result	Reference range
Red blood cell count (RBC)	3.68 ×10¹²/L	4.50–5.50 ×10¹²/L
Haemoglobin (Hb)	115 g/L	130–170 g/L
Haematocrit (Hct)	0.34 L/L	0.40–0.50 L/L
Mean corpuscular volume (MCV)	91.0 fL	83.0–101.0 fL
Mean corpuscular haemoglobin (MCH)	31.3 pg	27.0–32.0 pg
Platelet count	266 ×10⁹/L	150–400 ×10⁹/L
White blood cell count (WBC)	12.3 ×10⁹/L	4.0–10.0 ×10⁹/L
Neutrophils (absolute)	10.5 ×10⁹/L	2.0–7.0 ×10⁹/L
Lymphocytes (absolute)	0.8 ×10⁹/L	1.0–4.0 ×10⁹/L
Monocytes (absolute)	0.9 ×10⁹/L	0.2–1.0 ×10⁹/L
Eosinophils (absolute)	0.0 ×10⁹/L	<0.4 ×10⁹/L
Basophils (absolute)	0.1 ×10⁹/L	<0.1 ×10⁹/L
Sodium	135 mmol/L	132–144 mmol/L
Potassium	4.5 mmol/L	3.6–5.1 mmol/L
Chloride	90 mmol/L	94–110 mmol/L
Blood urea nitrogen (BUN)	34 mg/dL	8.4–25.7 mg/dL
Creatinine	1.2 mg/dL	0.7–1.3 mg/dL
C-reactive protein (CRP)	6 mg/L	<5 mg/L

The constellation of acute hyperactive delirium, severe psychomotor agitation, bilateral mydriasis, diffuse cutaneous flushing, dry skin, tachycardia, and severe hypertension raised the suspicion of an anticholinergic toxidrome. Careful medication reconciliation revealed that atropine ophthalmic solution had been prescribed for off-label sublingual administration to control refractory sialorrhea. Further history obtained from the patient's sister, who was his primary caregiver, disclosed marked visual impairment, preventing her from accurately counting the prescribed number of drops. Instead, she administered an unquantified number of drops directly onto the oral mucosa three times daily, stopping only when instructed by the patient. Consequently, the total atropine dose administered could not be estimated. This history, together with the characteristic clinical findings, strongly supported the diagnosis of accidental atropine-induced anticholinergic syndrome secondary to unintentional medication administration error.

**Figure 3:**
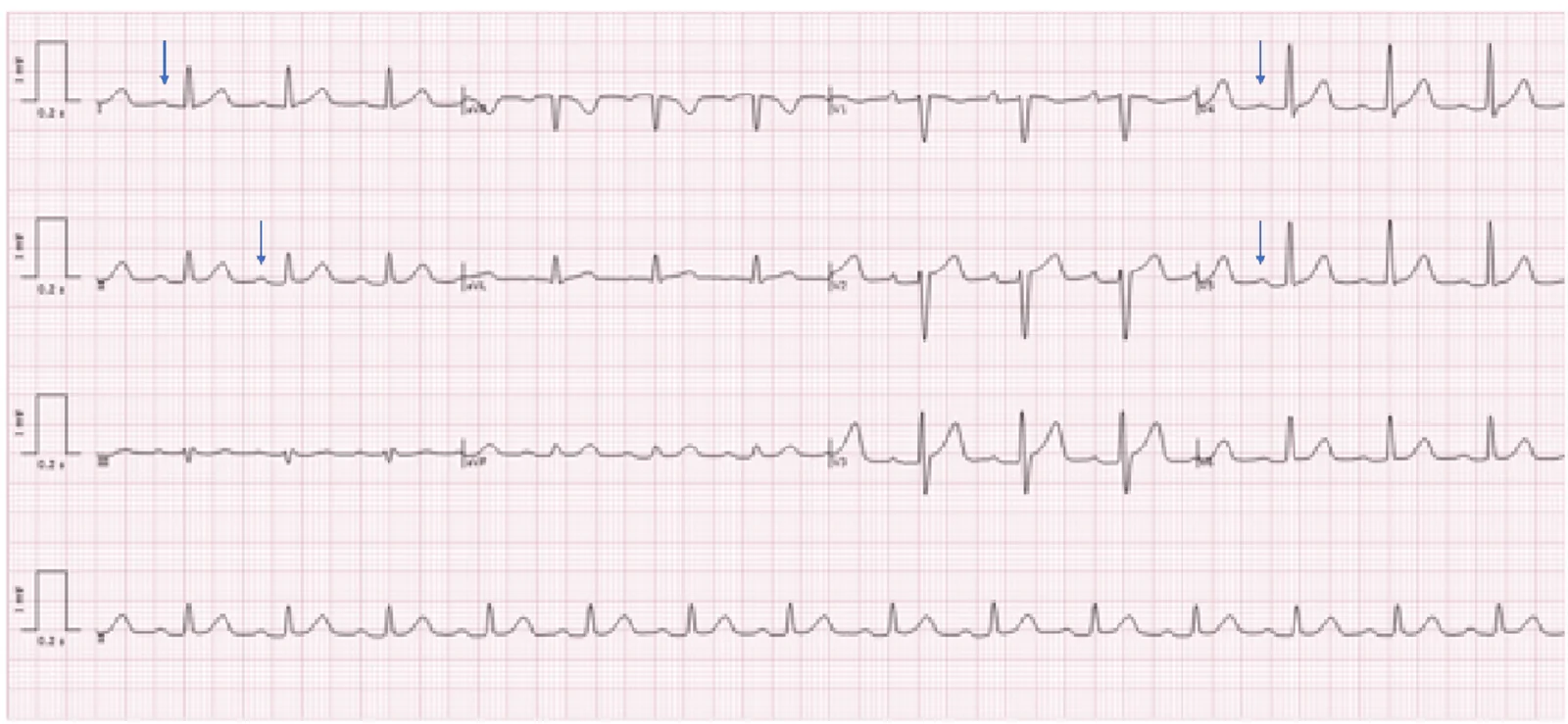
Follow-up 12-lead electrocardiogram after treatment The sinus rhythm was 87 beats/min, with P waves preceding each QRS complex, as indicated by the arrows. The automated corrected QT interval was 428 ms.

The Portuguese Poison Information Centre (Centro de Informação Antivenenos) was consulted and recommended continuous cardiovascular monitoring, supportive care, and intravenous neostigmine, because physostigmine was unavailable. The patient received 0.5 mg intravenous neostigmine, together with intravenous diazepam for agitation and antihypertensive treatment with intravenous labetalol followed by oral amlodipine for persistent hypertension. He remained under continuous observation, with progressive resolution of both neurological and autonomic manifestations (Figure 1C). Repeat electrocardiography demonstrated restoration of sinus rhythm at 87 beats/min with normalisation of the QTc interval to 428 ms (Figure 3). After approximately 14 hours of observation, the patient had returned to his neurological baseline and was discharged home. Sublingual atropine was discontinued and replaced with oral butylscopolamine for secretion control.

Subsequent oncology follow-up documented no recurrence of anticholinergic symptoms or neurological sequelae. Although atropine remained discontinued and butylscopolamine was continued, the available follow-up records did not specifically document subsequent control of sialorrhea.

## Discussion

This case illustrates a rare but clinically important presentation of atropine-induced anticholinergic syndrome secondary to an unintentional medication administration error during off-label sublingual atropine therapy. Although systemic toxicity following sublingual atropine has previously been reported, the available literature is limited and predominantly describes patients with neurological disorders receiving treatment for sialorrhea. Our case broadens the existing literature by describing severe toxicity in a patient with recurrent head and neck squamous cell carcinoma receiving palliative systemic therapy. More importantly, it highlights medication administration error as the principal mechanism of toxicity, reinforcing that preventable adverse drug events remain an important cause of acute medical presentations, particularly in older patients with complex multimorbidity receiving home-based care [5,10].

The development of systemic toxicity was most likely multifactorial and illustrates how human factors may interact with off-label prescribing to produce preventable adverse drug events. In this case, the combination of caregiver visual impairment, the need to administer an ophthalmic formulation requiring accurate drop counting, and home-based medication administration created conditions that favoured repeated unintentional overdose. Ophthalmic atropine formulations require the administration of a very small volume and, when used sublingually, are rapidly absorbed through the oral mucosa, allowing clinically significant systemic antimuscarinic effects after repeated supratherapeutic dosing [10,11]. Severe visual impairment of the primary caregiver prevented accurate counting of individual drops, resulting in repeated administration three times daily instead of the prescribed regimen. These findings demonstrate how apparently minor medication administration errors may lead to severe systemic toxicity when therapies requiring precise dose measurement are used in the home setting [2,10].

The diagnosis of anticholinergic syndrome remains primarily clinical and relies on recognition of a characteristic toxidrome rather than confirmatory laboratory investigations [3,10]. In our patient, the combination of hyperactive delirium, bilateral mydriasis, diffuse cervicothoracic flushing, hot dry skin, marked tachycardia, and severe hypertension was highly suggestive of systemic antimuscarinic toxicity. Importantly, these findings were interpreted within the clinical context of advanced head and neck cancer with a permanent tracheostomy, immediately raising the possibility of recently prescribed antisecretory therapy. Targeted medication reconciliation rapidly identified sublingual atropine as the causative agent. Alternative causes of acute delirium, including infection, metabolic disturbances, and other medication-related toxic syndromes, were considered but were not supported by the laboratory findings, medication history, or characteristic clinical features. The rapid and complete resolution of symptoms following atropine withdrawal and supportive treatment further reinforced the diagnosis. This case underlines the value of integrating physical examination, medication review, and clinical context when evaluating acute delirium in older adults with multimorbidity, in whom medication-related toxicity should always remain an important diagnostic consideration [3,10].

Management of anticholinergic syndrome is based on prompt discontinuation of the offending agent, supportive care, and close cardiorespiratory monitoring [3,10]. Benzodiazepines remain the preferred treatment for severe agitation, while physostigmine is considered the antidote of choice for carefully selected patients with severe central anticholinergic toxicity when no contraindications exist [3]. Because physostigmine was unavailable at our institution, intravenous neostigmine was administered following consultation with the Portuguese Poison Information Centre. Although neostigmine has limited penetration across the blood-brain barrier and therefore cannot be considered an equivalent substitute for physostigmine, it was considered a reasonable therapeutic option in this case given its availability and the patient's prominent peripheral anticholinergic manifestations. Our patient's rapid clinical improvement and complete neurological recovery reinforce that prompt recognition of the toxidrome, withdrawal of the offending agent, and appropriate supportive management remain the cornerstones of treatment [3,10].

This case also carries important implications for medication safety. Before prescribing off-label sublingual atropine, clinicians should ensure that both patients and caregivers are able to administer the medication accurately. Practical demonstration of the administration technique, together with confirmation of understanding, is particularly important when treatment is delivered by older caregivers or by individuals with visual, cognitive, or manual limitations [2,10]. When reliable dose administration cannot be guaranteed, alternative formulations or therapeutic options for sialorrhea should be considered [2,3]. Although serum atropine concentrations were not measured and the total administered dose could not be quantified, precluding assessment of a dose-toxicity relationship, the characteristic toxidrome, documented medication administration error, and complete clinical recovery following atropine withdrawal and supportive treatment provide strong evidence for the diagnosis. Ultimately, this case demonstrates that the safe use of off-label sublingual atropine depends as much on careful assessment and education of the caregiver as on appropriate prescribing.

## Conclusions

Sublingual atropine is an effective off-label option for the symptomatic management of refractory sialorrhea but may cause severe systemic anticholinergic toxicity when medication administration errors occur. This case highlights the importance of recognising the characteristic anticholinergic toxidrome, particularly in patients with advanced head and neck cancer receiving antisecretory therapy, where prompt medication reconciliation may rapidly identify the underlying cause. Careful assessment of the patient's and caregiver's ability to administer ophthalmic atropine accurately should form part of routine prescribing practice, especially when treatment relies on precise drop counting. Appropriate education, clear administration instructions, and consideration of alternative therapeutic strategies when accurate dosing cannot be ensured are essential to minimise preventable adverse drug events.
